# How does surgery compare to sham surgery or physiotherapy as a treatment for tendinopathy? A systematic review of randomised trials

**DOI:** 10.1136/bmjsem-2019-000528

**Published:** 2019-04-24

**Authors:** Dimitrios Challoumas, Christopher Clifford, Paul Kirwan, Neal L Millar

**Affiliations:** 1 Institute of Infection, Immunity and Inflammation, College of Medicine, Veterinary and Life Sciences, University of Glasgow, Glasgow, UK; 2 Department of Physiotherapy, NHS Greater Glasgow and Clyde, Glasgow, UK; 3 School of Physiotherapy, Royal College of Surgeons in Ireland, Dublin, Ireland; 4 Physiotherapy Department, Connolly Hospital Blanchardstown, Blanchardstown, Ireland

**Keywords:** tendinopathy, surgery, tendon, physiotherapy, sham surgery

## Abstract

**Purpose:**

To assess the effectiveness of surgery on all tendinopathies by comparing it to no treatment, sham surgery and exercise-based therapies for both mid-term (12 months) and long-term (> 12 months) outcomes.

**Methods:**

Our literature search included EMBASE, Medline, CINAHL and Scopus. A combined assessment of internal validity, external validity and precision of each eligible study yielded its overall study quality. Results were considered significant if they were based on strong (Level 1) or moderate (Level 2) evidence.

**Results:**

12 studies were eligible. Participants had the following types of tendinopathy: shoulder in seven studies, lateral elbow in three, patellar in one and Achilles in one. Two studies were of good, four of moderate and six of poor overall quality. Surgery was superior to no treatment or placebo, for the outcomes of pain, function, range of movement (ROM) and treatment success in the short and midterm. Surgery had similar effects to sham surgery on pain, function and range of motion in the midterm. Physiotherapy was as effective as surgery both in the midterm and long term for pain, function, ROM and tendon force, and pain, treatment success and quality of life, respectively.

**Conclusion:**

We recommend that healthcare professionals who treat tendinopathy encourage patients to comply with loading exercise treatment for at least 12 months before the option of surgery is seriously entertained.

**What is already known?**

- Much debate surrounds the role of surgical intervention in chronic tendon disease. Sham surgery trials are the gold standard against which to judge the effect of surgery on clinical conditions (such as tendinopathy).

**What are the new findings?**

- In 12 eligible randomised controlled trials in patients with various tendinopathies, surgery was not superior to sham surgery in patients with tendinopathy in the midterm and long term.
- Tendon loading exercises are as effective as surgery both in the midterm and long term for patients’ pain, function and quality of life.
- Surgery should be reserved for selected cases and only after a sufficiently long course (12 months) of evidence-based loading exercise has failed.

## Introduction

Tendinopathy poses a substantial socioeconomic burden globally comprising 30% of all general practice musculoskeletal consultations.^1^ Its aetiology is multifactorial and its exact pathophysiology remains uncertain; however, it appears to result from an imbalance between the protective/regenerative changes and the pathological responses that result from tendon overuse.^2 3^ The the most common exacerbating factor is thought to be overuse (particularly during sporting activities) causing repetitive microtrauma and consequent degeneration due to failure of the healing process.^4^ The net result is tendon degeneration, weakness, tearing, and pain.^5^

As the research on the management of tendinopathy is constantly increasing, new treatment modalities continuously emerge making decisions difficult for the treating healthcare professionals.^6^ In the absence of complete tendon tears, loading remains the mainstay of treatment and it is recommended as first line for all tendinopathies for 6 months.^7 8^ The choice of second-line treatment, which ranges from non-invasive modalities such as extracorporeal shock wave therapy (ESWT), glyceryl trinitrate patches^9^ and injection therapies to invasive surgery remains controversial.^10 11^

Surgery, which may be open or arthroscopic, is usually reserved for patients whose symptoms persist despite conservative management and complete tendon tears; however, its effectiveness has been repeatedly questioned.^6 12^ While expert opinion,^13 14^ guidelines^15 16^ and systematic reviews^17 18^ have attempted to provide guidance to the practising clinician on when surgery may be an appropriate next step the actual evidence from studies comparing surgical and non-surgical treatments on tendinopathies remains limited, and therefore definitive conclusions about the benefits and ideal timing of surgical intervention are yet to be reached.

Studies assessing the effectiveness of surgery in orthopaedics have had bias due to the inability for blinding.^19 20^ In recent years, studies have compared some orthopaedic operations (including surgery for tendinopathy) with sham surgery^21–23^ in a double-blinded manner to mirror the placebo effect of surgery. In those studies, there were no differences between control and intervention groups.^21–23^

The aim of this systematic review was to consider evidence that derives from studies assessing the effectiveness of surgery for tendinopathy in the general population. This includes comparisons of surgery (open or arthroscopic) with either non-surgical treatment modalities, sham surgery or no treatment in all tendinopathies with respect to the following outcome measures: pain, function, range of movement (ROM), force/strength, patient satisfaction, treatment success, quality of life (QoL) and complications.

## Methods

**Figure 1:**
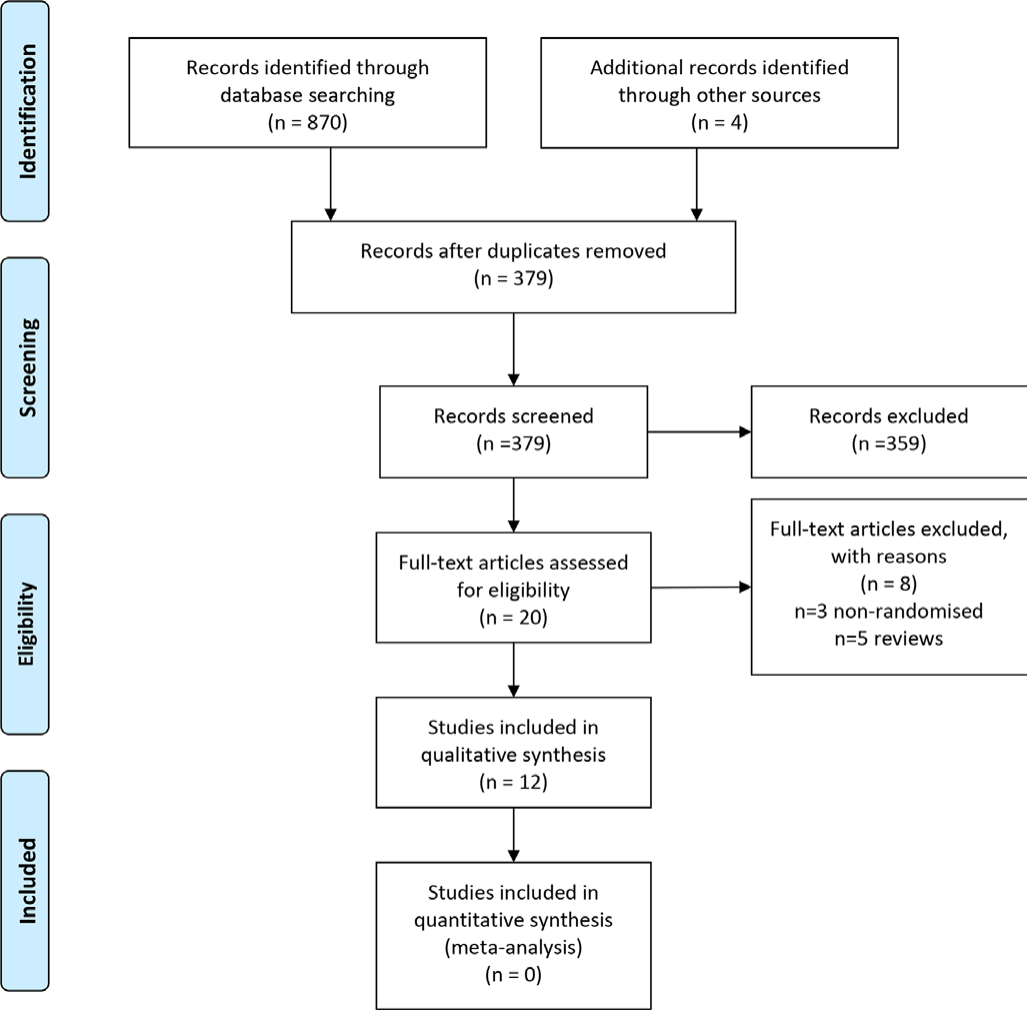
PRISMA flow diagram of included studies. PRISMA, Preferred Reporting Items for Systematic Reviews and Meta-Analyses.

The present systematic review has been conducted and authored according to the **‘**Preferred Reporting Items for Systematic Reviews and Meta-Analyses’^24^ (PRISMA) guidelines (figure 1).

### Eligibility

Included studies had a randomised design and compared surgery to any mode of non-surgical management for any type of tendinopathy in terms of at least one of the following outcomes: ‘pain’, ‘function’, ‘ROM’, ‘force/strength’, ‘patient satisfaction’, ‘treatment success’, ‘QoL’, ‘complications’. Non-randomised observational studies, case reports, case series and literature reviews were excluded. Participants had to be over 18 years of age with a clinical diagnosis of tendinopathy with or without radiological signs. Studies including patients with full tendon tears were excluded. Duration of symptoms/signs was not a criterion, neither was length of conservative treatment and follow-up. Language criterion was not applied.

### Search strategy

A thorough literature search was conducted by two of the authors (DC and CC) independently via Medline, EMBASE, Scopus and CINAHL in March 2018, with the following Boolean operators: ‘(tendinopathy OR tendinosis OR tendinitis OR tendonitis OR tennis elbow OR jumper’s knee OR lateral elbow tendinopathy OR lateral epicondylitis OR rotator cuff disease OR shoulder impingement OR patellar OR Achilles) AND (surgery OR surgical management OR surgical treatment OR tenotomy OR open surgery OR arthroscopic surgery) AND (conservative management OR conservative treatment OR physiotherapy OR eccentric exercises OR eccentric strengthening OR stretching OR shock-wave therapy OR ESWT OR extracorporeal shock wave therapy OR ultrasound OR iontophoresis OR laser OR LLLT OR polidocanol OR sclerotherapy OR botox OR botulinum toxin OR GTN OR glyceryl trinitrate OR nitroglycerin OR corticosteroid injections OR platelet rich plasma OR PRP OR autologous blood OR sham surgery)’.

Medical Subject Heading terms were not used to minimise the risk of missing relevant articles. Review articles were used to identify eligible articles that were missed at the initial search. Additionally, reference list screening and citation tracking in Google Scholar were performed for each relevant article.

### Screening

From a total of 874 articles that were initially identified, after exclusion of duplicate and non-eligible articles, title and abstract screening and addition of missed studies identified by review articles, reference list screening and citation tracking, 12 studies were found to fulfil the eligibility criteria. Figure 1 illustrates the article screening process according to PRISMA guidelines.^24^

### Quality assessment

For a thorough assessment of the studies, internal validity (freedom from bias), external validity (generalisability/applicability) and precision (reproducibility/freedom from random error) were all assessed separately by two of the authors (DC and CC) independently and a third independent opinion (PK) was sought where disagreements existed. Quality scales and resulting scores were not used as these usually combine aspects of study methodology with aspects of reporting; therefore, they are thought to be inappropriate for assessment of study quality.^25^ In addition, score cut-offs classifying studies of good or poor quality are usually not provided and consequently these are usually made up by the author of the review article which can be highly variable.

For internal validity, the ‘Cochrane Collaboration’s tool for assessing risk of bias in randomised trials’ was used, which includes six questions/criteria assessing the risk of five specific and one non-specific (‘other’) types of bias.^25^ As ‘other’ bias, our preset assessment criteria were as follows: (a) adequate and appropriate inclusion and exclusion criteria, (b) differences between treatment and control groups at baseline (confounding) and (c) appropriateness of statistical tests deployed. External validity was assessed based on the population, age range and clinical relevance of interventions and outcome measures. For the assessment of precision, the sample size, performance of statistical power calculation and p values that were used to define statistical significance were taken into account.

In the Cochrane Collaboration’s tool, each item is classified as ‘high’, ‘low’ or ‘unclear’ risk of bias. No total scores are given. External validity and precision of each study were rated separately as of ‘high’, ‘low’ or ‘unclear’ risk.

Overall, studies were characterised as of ‘good’, ‘moderate’ or ‘poor’ quality based on a combined assessment of their internal validity, external validity and precision which was again conducted by two of the authors independently (DC and CC) and the opinion of a third author was provided where the two judgements differed. The criteria used for overall quality assessment were as follows: ‘Good’-quality studies had ‘high’ risk of bias in <2 of the internal validity categories, external validity and precision; ‘Moderate’-quality studies had ‘high’ risk bias in two of the internal validity categories, external validity and precision; ‘Poor’-quality studies had ‘high’ risk of bias in >2 of the internal validity categories, external validity and precision.

### Data extraction: handling

Each of the eligible articles was initially read by the first author to gain familiarity and subsequently each article was re-read and their key characteristics were extracted and inserted in tables in Microsoft Word to facilitate analysis and presentation.

For the presentation of results, outcomes were divided into midterm (up to 1-year follow-up) and long term (more than 1-year follow-up). Where results were reported at more than one time points in the midterm and in the long term, the longest-term results were used for each study in the results tables; however, findings at all follow-up stages are described in text in the results section. Where studies used tools and questionnaires as part of outcome measures, their results were tabulated under the generic outcome category according to the aim of the questionnaire. Where results of their specific subcomponents were presented too, additional results were tabulated under the corresponding outcome category: for example, where the Oxford Shoulder Score was used, the aim of which is functional assessment, results of the overall score were used for ‘function’; if the findings of specific questions of the questionnaires that are related to ‘pain’ were also described, this specific result was also used for ‘pain’, etc. The outcome category ‘complications’ included all generic and surgery-specific intraoperative and postoperative complications as well as progression of disease to full tendon tears and other debilitating conditions (eg, adhesive capsulitis).

To classify the strength of evidence for each outcome reported, we used the rating system formulated by Van Tulder *et al*,^26^ which consists of four levels of evidence: strong evidence (Level 1) is provided by generally consistent findings in multiple high-quality randomised controlled trials (RCTs). Moderate evidence (Level 2) is provided by generally consistent findings in one high-quality RCT and one or more low-quality RCTs, or by generally consistent findings in multiple low-quality RCTs. Limited or conflicting evidence (Level 3) is provided by only 1 RCT (either high or low quality), or by inconsistent findings in multiple RCTs. No evidence (Level 4) is defined by the absence of RCTs.

As our overall quality assessment included a ‘moderate’-quality category, we extended Level 2 to ‘evidence provided by generally consistent findings in high-quality RCT and one or more low-quality or moderate-quality RCTs or multiple-moderate-quality RCTs’. Two of the authors (DC and CC) jointly decided on the level of evidence for each outcome based on the aforementioned system without any disagreements. Results were considered to be significant when they were based on either strong or moderate evidence.

### Definitions and acronyms

Physiotherapy (any tendon rehabilitation regime administered regularly aiming to strengthen the affected tendon includes ‘supervised exercises’ and ‘eccentric training’; does NOT include standard postoperative rehabilitation); sham surgery (a faked surgical intervention that omits the step thought to be therapeutically necessary); ORI-TETS (the Orthopaedic Research Institute Tennis Elbow Testing System); OSS (Oxford Shoulder Score); SDQ (Strengths and Difficulties Questionnaire); HADS (Hospital Anxiety and Depression Score); VAS (Visual Analogue Scale); EQ VAS (EuroQoL VAS); EQ-5D-3L (EuroQoL 5 Dimensions 3 Level index); PRIM (Project on Research and Intervention in Monotonous work); QoL (Quality of Life); UCLA (University of California Los Angeles score); VISA (Victorian Institute of Sport Assessment); ROM: range of movement; 15D (15-dimensional).

## Results

A total of 12 eligible studies were identified with a total of n=1051 participants (mean 87.4±80.9) with n=1056 affected tendons (five bilateral); of these, n=459 tendons had surgery, n=258 tendons received non-surgical treatments (n=178 physiotherapy, n=50 ESWT, n=30 placebo laser, n=20 botox, n=10 polidocanol), n=116 had sham surgery (placebo), n=30 had detuned laser (placebo) and n=104 had observation only (no treatment). Treatment was considered to be combined (surgery +physiotherapy) in three studies, wherein it was specifically stated that the postoperative physiotherapy was the same as or similar to the regime administered to the physiotherapy only group.^27–29^ Patients treated with surgery in all other studies followed a standard postoperative rehabilitation programme. Affected tendons had one of shoulder tendinopathy (n=876), lateral elbow tendinopathy (n=122), patellar tendinopathy (n=40) or Achilles tendinopathy (n=20). Of the tendons treated surgically (including sham surgery), n=177 operations were performed open and n=398 arthroscopically. Surgery in those with lateral elbow tendinopathy, Achilles and patellar tendinopathy was open in all cases while that for shoulder tendinopathy was either open (n=45) or arthroscopic (n=398). A total of eight studies were controlled as at least one of their treatment groups received either placebo (detuned laser or sham surgery) or an exercise regime which has repeatedly been proven to be effective and is currently recommended as first-line treatment for all tendinopathies. Mean age was 48.0 years (range 18–72). All studies included patients with chronic tendinopathy (duration of symptoms >3 months). Length of follow-up varied from 6 months to >10 years (median 12 months). Publication years ranged from 1993 to 2018.

Table 1 shows the methodological characteristics and table 2 presents the summary of samples, interventions and outcome measures of the included studies.

**Table 1:** Methodological characteristics of included studies

First author— tendinopathy	Study type	Randomisation method	Blinding method	Allocation concealment	Statistical power calculation	Baseline comparison	Inclusion criteria	Exclusion criteria	Follow-up completion
Brox^36^— Shoulder	Randomised controlled trial	Random permuted blocks	Patients wearing t-shirts at follow-up to hide scar; patients asked not to talk to assessor about treatment	–	Yes, 90%	Less women in surgery group	Age 18–66 y, shoulder pain for >3 m, resistant to physio and drugs, dysfunction/pain on abduction, normal passive ROM, pain during two of the three isometric-eccentric tests, positive impingement tests	Acromioclavicular joint arthritis, cervical syndrome, rotator cuff rupture, glenohumeral instability, bilateral muscular pain with tenderness and severely decreased ability to relax the shoulder/neck/temporomandibular joint on examination, reluctant to accept one or more of the treatment regimens of the study	99%
Rahme^37^ —Shoulder	Randomised controlled trial	Blocked randomisation	Independent assessor	–	No	No comparison	Isolated shoulder disease, working age, shoulder pain >1 y at rest accentuated by movements involving elevation, positive Hawkins (impingement) sign, positive impingement test (relief of symptoms within 15 min of injection of local anaesthetic)	Glenohumeral osteoarthritis, those requiring resection of the lateral end of the clavicle	93%
Rompe^30^— Shoulder	Randomised trial (non-controlled)	Based on whether reimbursement for ESWT was approved by insurance company	Not blinded	–	–	No differences	Calcareous deposit on standard AP radiographs of a diameter of at least 10 mm; the morphological features of the deposit had to be homogeneous in appearance and with well-defined borders, or inhomogeneous in structure with sharp outline or homogeneous in structure with no defined border, shoulder pain for more than 12 m, clinical signs of subacromial impingement, unsuccessful conservative therapy in the previous 6 m, no evidence of bone-related anatomic outlet impingement or functional outlet impingement as seen on radiographs or MRI	Cloudy and transparent appearance of deposit, radiological signs of spontaneous resorption, evidence of type III acromial morphological feature according to Bigliani *et al* on the outlet view of the acromion, evidence of subacute acromial bursitis, evidence of acromial spur or acromioclavicular osteophytes on AP radiographs, evidence of rotator cuff tears on MRI, evidence of functional impingement of rotator cuff on USS or arthroMRI, tears of the glenohumeral ligaments of the labrum, hypertrophy of supraspinatus muscle, dysfunction in neck or thoracic region, prior shoulder surgery, local degenerative disease of shoulder, RA, neurological abnormalities of the upper limb with calcifying tendinitis, pregnancy, infection, tumour	75%
Haahr^35^— Shoulder	Randomised controlled trial	Computer-generated random sequence generation	Not blinded	Sealed envelopes	Yes, 80%	More sick leave due to more severe shoulder pain in surgery group	Fulfilment of all diagnostic criteria (shoulder pain, pain on abduction with painful arc, Hawkins sign, positive impingement test (relief of symptoms within 15 min of injection of local anaesthetic), symptoms 6 m–-3 y, age 18–55 y, normal passive glenohumeeal movements	Impaired glenohumeral rotation, history of acute trauma, previous surgery of fracture near affected shoulder, known OA in glenohumeral or acromioclavicular joint, clacifications >2 cm in rotator cuff tendons, rupture of the cuff, cervical root syndromes	93%
Ketola^29^ — Shoulder	Randomised controlled trial	Computer-generated numbers	Independent physiotherapist conducted 5-year assessment; participants wearing t-shirts to cover scars and asked not to indicate treatment group	Randomisation by independent statistician; sealed envelopes	Yes, 80%	No difference in outcome measures, no comparison of demographics	Clinical symptoms of shoulder impingement, positive Neer’s test, symptoms for at least 3 m, failed management with rest, NSAIDs, steroid injections and regular physiotherapy, age 18–60 y, no previous shoulder operations, willingness and capacity to comply with study protocol	OA, glenohumeral instability, penetrating rotator cuff rupture, cervical radiculopathy, adhesive capsulitis, shoulder neuropathy	78%
Farfaras^28^—Shoulder	Randomised controlled trial	Envelopes divided in boxes based on sex and age	Independent physiotherapist conducted assessment; participants encouraged to wear a t-shirt at follow-up to conceal their scar	–	Yes, sample size not enough for 80% power	No difference	Positive Neer and Hawkins tests, failed conservative management, subacromial pain for more than 6 m	Diabetes mellitus, neurological or spine disorders, radiographic OA, chronic joint disorders, full-thickness rotator cuff tear, subacromial impingement syndrome stage 3	63%
Beard^34^— Shoulder	Randomised controlled trial	Automated computer-generated minimisation system	Double blinded except ‘no treatment’ group; masked assessment	Centralised telephone randomisation centre used	Yes, 90%	No difference	Subacromial pain for at least 3 m, consultant’s clinical diagnosis, eligible for arthroscopic surgery, completion of conservative management programme including physiotherapy and at least one steroid injection	Full-thickness rotator cuff tear, other shoulder pathology identified on MRI or USS, previous shoulder surgery on affected side, RA or other inflammatory joint conditions, cervical spine pathology, previous septic arthritis in shoulder, radiotherapy in same side as affected shoulder, lacking consent, cognitive impairment or language issues, unable to perform clinical assessments, >75 y of age	81%
Bahr^27^— Patella	Randomised controlled trial	Randomisation sequence in blocks of four created by statistician; those who had failed eccentric strengthening were allocated to secondary surgery group	Not blinded	Sealed envelopes; randomisation sequence created by statistician	Yes, 90%	No differences	History of exercise-related pain in proximal patellar tendon or patellar insertion and tenderness to palpation, pain during and after activity and unable to participate in sports at same level as before onset of pain, thickening and increased signal intensity on MRI	History of knee/patellar tendon surgery, inflammatory or degenerative joint condition, less than 18 y of age, inability to understand oral and written Norwegian	88%
Alfredson^33^— Achilles	Randomised trial (non-controlled)	Box with envelopes	Not blinded	Opaque envelopes	No	No comparison as information not provided	–	–	95%
Radwan^31^— Lateral elbow	Randomised trial (non-controlled)	Closed envelopes	Not blinded	Closed envelopes	Yes, 80%	Characteristics of two groups presented but comparison not performed	Established diagnosis of lateral epicondylitis with failure of conservative Tx for 6 m (NSAIDs, steroid injections, physical therapy, exercise programme, elbow brace) Pain induced by >1 of palpation of lateral epicondyle, resisted wrist extension, chair test	Younger than 18 y, local infection, malignancy, elbow arthritis, generalised polyarthritis, ipsilateral shoulder dysfunction, neurological abnormalities, radial nerve entrapment, cardiac arrhythmia, pacemaker, steroid injection last 6 w, pregnancy	89%
Kroslak^22^— Lateral elbow	Randomised controlled trial	Computer-generated code	Double blinded	Sealed, unmarked envelopes	Yes, 90% but not enough participant recruited		>18 y of age, clinical diagnosis lateral epicondylitis (point tenderness over lateral epicondyle and worse pain with chair pick-up test and maximal hand grip), failed conservative therapy for 6 m (including injections)	Previous surgery or dislocation of affected elbow, steroid injection in last 3 m, inadequate skin coverage over elbow, sensory/motor changes distal to elbow, unwillingness/inability to attend follow-up or enter either treatment arm	85% (42% for clinical assessment)

AP, antero-posterior;ESWT, extracorporeal shock wave therapy;m, months; MRI; magnetic resonance imaging; NSAIDs, non-steroidal anti-inflammatory drugs;OA, osteoarthritis;RA, rheumatoid arthritis;ROM, range of movement;USS, ultrasound scan;w, weeks;y, years.

**Table 2:** Description of samples, interventions and outcome measures

First author	Tendon affected	Min duration of symptoms	Sample, mean/median age (range), %F	Interventions	Supervision? (physio only)	Follow-up	Outcome measures
Brox^36^	Rotator cuff	3 m	N=125; mean 48 y (18–66 y); 47%	Arthroscopic surgery (n=45) or physiotherapy (n=50) or detuned laser (placebo) (n=30)	Yes, 3–6 m	3 m, 6 m, 2.5 y	(a) Neer shoulder score (pain during previous week 0–35, function tests (muscle tests, reaching ability, stability 0–30 and active ROM 0–25), anatomical/radiological evaluation 0–10), (b) pain at rest, at night, during activity during previous week (1–9), (c) emotional distress with Hopkins symptom checklist (0–25), (d) costs
Rahme^37^	Rotator cuff	12 m	N=42; mean 42 y (28–63 y); 55%	Open surgery (n=21) or physiotherapy (n=21)	Yes, not stated	6 m, 12 m	(a) VAS for pain at rest plus VAS for pain during ‘pour out of pot’ manoeuvre (treatment success if >50% improvement compared with baseline)
Rompe^30^	Rotator cuff	12 m	N=79; mean 50.8 y (31–68 y); 61%	ESWT 3000 impulses, 0.6 mJ/mm^2^(n=50) or open surgery (n=29)		12 m, 24 m	(a) UCLA rating for pain and function of the shoulder (Kay and Amstutz): max score 35 points; pain 1–10, function 1–10, active range forward flexion 0–5, strength in forward flexion 0–5, patient satisfaction 0–5, (b) Outcomes score (>33 excellent, 29–33 good,<29 poor), (c) radiological evaluation: AP radiograph 1 day before surgery or ESWT and at 12 m; resorption graded as none, partial or complete
Haahr^35^ Hahr (2009)	Rotator cuff	6 m	N=90; mean 44.4 y(; 69%	Arthroscopic surgery (n=45) or physiotherapy (n=45)	Yes, 12 w	3 m, 6 m, 12 m, >4 y	(a) Constant score (primary outcome; 0–100; includes VAS for pain, limitations in ADLs, active ROM of glenohumeral joint in four directions, isometric shoulder strength measurement), (b) Likert scale (0–9, numerical box complaint scale), (c) employment within last 3 m, (d) sick leave, (e) labour compensation claims
Ketola^29^	Rotator cuff	3 m	N=140; mean 47.1 y (23–60 y); 63%	Arthroscopic surgery +physiotherapy (n=70) or physiotherapy (n=70)	Yes, mean 6.5 visits	3 m, 6 m, 12 m, 24 m, 60 m	(a) VAS for pain (0–10; primary measure), (b) VAS for disability (0–10), (c) VAS for working ability (0–10), (d) VAS for pain at night (0–10), (e) SDQ score, (f) number of painful days in previous 3 m, (g) proportion of pain-free patients (VAS for pain <4), (h) health-related QoL (15-day tool) at 5 y
Farfaras^28^, Farfaras (2018)	Rotator cuff	6 m	N=87; mean 49.3 y (41–78); 51%	Arthroscopic surgery +physiotherapy (n=29) or open surgery +physiotherapy (n=24) or physiotherapy (n=34)	Yes, 3–6 m	2.5 y, >10 y	(a) Constant score (0–100; includes VAS for pain, limitations in ADLs, active ROM of glenohumeral joint in four directions, isometric shoulder strength measurement), (b) SF-36 (general health), (c) Watson & Sonnabend score (0–3, 14 questions), (d) ROM active elevation and internal rotation, (e) abduction strength, (f) USS and X-ray both shoulders
Beard^34^	Rotator cuff	3 m	N=313; mean 53 y 50%	Arthroscopic surgery (n=106) or sham surgery (n=103) or no treatment (n=104)		6 m, 12 m	(a) OSS (primary outcome; 0–48), (b) modified Constant-Murley Shoulder Score (for function and ROM), (c) Pain DETECT (questionnaire for neuropathic pain), (d) Quantitative sensory testing, (e) adverse events, (f) QoL life (EQ-5D-3L), (g) EQ VAS, (h) treatment expectations, (i) patient perception or satisfaction, (j) anxiety and depression (HADS score).
Bahr^27^	Patellar	3 m	N=35 (40 tendons); mean 31 y (19–49 y); 14%	Open surgery +physiotherapy (n=20 tendons) or physiotherapy (n=20 tendons)	Yes, 12 w	3 m, 6 m, 12 m	(a) VISA score (0–100), (b) global evaluation score (−5 to +5), (c) treatment satisfaction (4-grade scale), (d) functional tests (standing jumps, counter-movement jumps and leg extension strength), (e) VAS score for pain after each functional test (0–10)
Alfredson^33^	Achilles	6 m	N=20; mean 46 y; 55%	Open surgery (n=10) or polidocanol injection (n=10)		12 w, 6 m	(a) VAS for pain (0–100) during activity, (b) patient satisfaction (satisfied or not satisfied)
Radwan^31^	Wrist extensors	6 m	N=56; mean 40 y (22–60 y); 41%	ESWT 1800 impulses, 0.22 mJ/mm^2^ (n=29) or open surgery (n=27)		3 w, 6 w, 12 w, 1 y	(a) Pain (at night, at rest) with VAS score (0–100), (b) residual pain at 12 m based on criteria by Roles & Maudsley (excellent, good, acceptable, poor), (c) tenderness with VAS score (0–100), (d) grip strength (scale 1–4), (e) treatment success: asymptomatic at 15 days
Kroslak^22^	Wrist extensors	6 m	N=26 mean 51.5 y (41–77 y); 68%	Open surgery (n=13) or sham surgery (n=13)		2 w, 6 w, 12 w, 26 w, >1 y	(a) Pain (frequency and severity) with Likert-based verbal descriptor scale with activity, at rest and during sleep, (b) Self-rated function (picking up objects, twisting motions, elbow stiffness, overall elbow rating), (c) point tenderness (Likert verbal descriptor pain scale), (d) elbow stiffness and ROM, (e) Maximal force of wrist extension during chair pick-up test using ORI-TETS, (f) maximal grip strength

ADLs, activities of daily living; AP, antero-posterior; EQ-5D-3L, EuroQoL 5 Dimensions 3 Level index; ESWT, extracorporeal shock wave therapy; HADS, Hospital Anxiety and Depression Score; ORI-TETS, Orthopaedic Research Institute Tennis Elbow Testing System; OSS, Oxford Shoulder Score; QoL, quality of life; ROM, range of movement; SDQ, Strengths and Difficulties Questionnaire; SF-36, Short Form Health Survey; UCLA, University of California Los Angeles score; USS, ultrasound scan; VAS, Visual Analogue Scale; VISA, Victorian Institute of Sport Assessment; m, months; w, weeks; y, years.

### Quality assessment

Table 3 illustrates our assessment of internal validity, external validity, precision and overall quality of each study. Six studies were found to be of ‘poor’ overall quality, four of ‘moderate’ quality and two of ‘good’ quality.

**Table 3:** Quality assessment of included studies

First author	Internal validity (Cochrane’s collaboration tool for assessing risk of bias)							External Validity	Precision	Overall quality
	*Selection bias*		*Performance bias*	*Detection bias*	*Attrition bias*	*Reporting bias*	*Other*			
	*Random sequence generation*	*Allocation concealment*	*Blinding of patients and staff*	*Blinding of outcome measures*	*Completeness of outcome data*	*Selective reporting*				
Brox^36^	Low	?	High	Low	Low	High	Low	Low	Low	Moderate
Rahme^37^	Low	?	High	Low	High	High	High	High	Low	Poor
Rompe^30^	High	?	High	High	Low	Low	Low	Low	?	Poor
Keizer^32^	?	?	High	High	Low	High	High	Low	High	Poor
Haahr^35^	Low	Low	High	High	Low	Low	Low	Low	Low	Moderate
Bahr^27^	Low	Low	High	High	Low	Low	Low	Low	Low	Moderate
Alfredson^33^	Low	Low	High	High	Low	High	High	High	High	Poor
Radwan^31^	?	Low	High	High	Low	Low	?	Low	Low	Moderate
Ketola^29^	Low	Low	High	Low	Low	High	?	High	Low	Poor
Farfaras^28^	Low	?	High	Low	High	Low	Low	Low	High	Poor
Beard^34^	Low	Low	Low High	Low	Low	Low	Low	Low	Low	Good
Kroslak^22^	Low	Low	Low	Low	Low	Low	Low	Low	High	Good

### Internal validity

#### Selection bias

All 12 studies were randomised. Nine (9) studies were thought to have ‘low’ risk of bias and one study was labelled as ‘high’ risk as randomisation was based on whether reimbursement for ESWT was approved by the insurance company.^30^ The randomisation method was not described in sufficient detail in two studies^31 32^ (‘unclear’ risk). Risk of bias with regard to allocation concealment was considered ‘low’ in seven studies wherein either randomisation was performed by an independent statistician, a centralised telephone randomisation centre or the authors specifically state that sealed/closed/opaque envelopes were used.^22 27 29 31 33–35^ The remaining five were classified as ‘unclear’ risk as details were not provided.

#### Performance bias

Patients were only blinded in the two studies that compared surgery with sham surgery.^22 34^ However, in the study by Beard *et al,*^34^ only the two of the three groups were blinded. As some patients received no treatment, the part of the study that compared the surgical groups to the no treatment group was rated as ‘high’ risk of bias; the part that compared the two surgical treatments was ‘low’ risk. In the remaining 10 studies, blinding of participants was not possible (surgery vs non-surgical treatment; ‘high’ risk).

#### Detection bias

Blinding of outcome measures was thought to be sufficient (‘low’ risk) in studies wherein attempts were made to blind the assessors by (a) using independent assessors, (b) asking the participants not to disclose the nature of their treatment to assessors and to (c) wear t-shirts to hide surgical scars were applicable.^22 28 29 34 36 37^ All other studies (n=6) were labelled as ‘high risk’.

#### Attrition bias

Reasons for dropouts/withdrawals of participants were adequately reported in all studies (‘low’ risk) but one^37^ (‘high’ risk). Rate of follow-up completion was considered of ‘high’ risk in the study by Farfaras *et al*,^28^ where it was only 63%. In the study by Kroslak & Murrell,^22^ follow-up completion rate was 85% for the self-rated outcomes but only 42% for the clinical tests; however, the study was rated as ‘low’ risk of bias as the primary outcome measure was self-rated (frequency of elbow pain during activity at 6 months).

#### Reporting bias

Reporting of results was found to be inappropriate or inadequate in five studies (‘high’ risk); Alfredson *et al*,^33^ Rahme *et al*^37^ and Ketola *et al*^29^ only included self-reported parameters in their outcome measures and additionally the first two studies only included VAS for pain (Rahme *et al*^37^) or VAS for pain and satisfaction (Alfredson *et al*^33^). Keizer *et al*^32^ used categorical variables in their analysis with an inappropriately small number of categories in some cases; for example, ROM was classified as either ‘normal’ or ‘limited (>5 degrees)’. Additionally, Alfredson *et al*^33^ did not include any graphical or tabular representation of their results. Brox *et al*^36^ and Alfredson *et al*^33^ did not present details, statistical comparisons or p values for some of their findings. The remaining six studies were rated as ‘low’ risk.

#### Other bias

Inclusion and exclusion criteria were thought to be adequate for all but two studies: Alfredson *et al*^33^ did not mention any eligibility criteria at all and the exclusion criteria of Rahme *et al*^37^ was limited to ‘glenohumeral osteoarthritis and those requiring resection of the lateral end of the clavicle’. Baseline characteristics of the treatment control groups were presented by all but two studies (‘high’ risk; Alfredson *et al*^33^ and Rahme *et al*^37^). Of the remaining 10 studies, one did not perform statistical analyses comparing the two groups at baseline (‘unclear’ risk; Radwan *et al*^38^), one only compared outcome measures and not demographics (‘unclear’ risk; Ketola *et al*^29^). Eight (8) studies performed adequate baseline comparisons; five of them reported no differences in demographics or outcome measures between treatment groups (‘low’ risk; Bahr *et al,*^27^ Beard *et al*,^34^ Farfaras *et al*,^28^ Kroslak & Murrell,^22^ Rompe *et al*^30^) and the other three found trivial differences that were regarded as introducing ‘low’ risk of bias (Brox *et al,*^36^ Haahr *et al*^35^ and Keizer *et al*^32^ (table 1). The risk of ‘other’ bias in the study by Keizer *et al*^32^ was classified as ‘high’ as some of the patients in their botox group received a second injection at 6 weeks follow-up and some others ended up having surgery. Appropriate statistical tests and comparisons were deployed in all studies except for Rahme *et al*^37^ who utilised a ‘as treated’ and not a ‘intention-to-treat’ basis when comparing groups at 12 months, although the authors themselves acknowledge this limitation in the manuscript.

### External validity

General, non-specific populations were used in all studies. Age ranges of participants were wide enough to allow for good generalisability in all studies. Clinically relevant assessment tools and outcome measures were used in nine studies. Alfredson *et al*^33^ and Rahme *et al*^37^ only included self-reported pain and satisfaction, whereas Ketola *et al*^29^ used a much greater number of measures, all of which were, however, also self-reported (‘high’ risk). The nature, frequency and intensity of physiotherapy that were considered appropriate were used, and no guidelines exist about the best formulation or dosage of the other non-surgical treatments (botox, polidocanol and ESWT) in clinical practice; therefore, all doses and frequencies used were considered clinically relevant (‘low’ risk).

### Precision

Statistical power calculation prior to recruitment was performed in all but three studies (Alfredson *et al,*^33^ Keizer *et al*^32^ and Rompe *et al*^30^). The studies by Alfredson *et al*^33^ and Keizer *et al*^32^ had small sample sizes (n=20 and n=40, respectively) in addition to their failure to perform statistical power calculation; therefore, they were rated as ‘high’ risk of bias. The study by Rompe *et al*^30^ was classified as ‘unclear’ risk as its much larger sample size (n=79) is comparable to studies that recruited to a power of at least 80%. Where a power calculation was performed, sample sizes were adequate for a power of at least 80% except for the study by Farfaras *et al*^28^ (‘high’ risk). Levels of significance were set at p=0.05 in all studies except for that of Alfredson *et al*^33^ where the level of significance is not stated.

### Findings of included studies

Tables 4a and b provide a summary of midterm (up to 1-year follow-up) and long-term (>1-year follow-up) results along with levels of evidence for the overall results of each outcome measure.

**Table 4a:** Mid-term results (<1-year follow-up)

Treatment modes	Tendon affected	First author (year)	Pain	Function	ROM	Strength	Satisfaction	Treatment Success	QoL	Complications
Surgery versus detuned laser or no treatment	Rotator cuff	Brox^36^	↓	↑	↑	–	–	↑	–	↔
	Rotator cuff	Beard^34^	↓	↑	↑	–	↑	↑	↑	↔
**Overall surgery versus detuned laser or no treatment (evidence level**)			**↓ (2**)	**↑ (2**)	**↑ (2**)	– (4)	↑ (3)	**↑ (2**)	↑ (3)	**↔ (2**)
Surgery versus sham surgery	Rotator cuff	Beard^34^	↔	↔	↔	–	↔	↔	↔	↔
	Wrist extensors	Kroslak^22^	↔	↔	↔	↔	–	–	–	↔
**Overall surgery versus sham surgery (evidence level**)			**↔ (2**)	**↔ (2**)	**↔ (2**)	↔ (3)	↔ (3)	↔ (3)	↔ (3)	**↔ (2**)
Surgery versus physiotherapy	Rotator cuff	Brox^36^	↔	↔	↔	–	–	–	–	–
	Rotator cuff	Rahme^37^	↔	–	–	–	–	–	–	–
	Rotator cuff	Haahr^35^	↔	↔	↔	↔	–	–	–	–
	Patellar	Bahr^27^	↔	↔	–	↔	↔	↔	–	–
**Overall surgery versus physiotherapy (evidence level**)			**↔ (2**)	**↔ (2**)	**↔ (2**)	**↔ (2**)	↔ (3)	↔ (3)	– (4)	– (4)
Surgery versus ESWT (evidence level)	Rotator cuff	Rompe^30^	↔ (3)	– (4)	– (4)	– (4)	– (4)	↔ (3)	– (4)	– (4)
Surgery versus botox (evidence level)	Wrist extensors	Keizer^32^	↔ (3)	– (4)	↔ (3)	↔ (3)	– (4)	↔ (3)	– (4)	– (4)
Surgery versus polidocanol (evidence level)	Achilles	Alfredson^33^	↔ (3)	– (4)	– (4)	– (4)	↔ (3)	– (4)	– (4)	– (4)

up arrow = increased; down arrow = decreased; side arrow = no changes

ESWT, extracorporeal shock wave therapy; QoL, quality of life.

**Table 4b:** Long-term results (>1-year follow-up)

Treatment modes	Tendon affected	First author (year)	Pain	Function	ROM	Force	Satisfaction	Treatment Success	QoL	Complications
Surgery versus detuned laser or no treatment (evidence level)	Rotator cuff	Brox^36^	– (4)	– (4)	– (4)	– (4)	– (4)	↑ (3)	– (4)	– (4)
Surgery versus sham surgery (evidence level)	Wrist extensors	Kroslak^22^	↔ (3)	↔ (3)	↔ (3)	↔ (3)	– (4)	– (4)	– (4)	↔ (3)
Surgery versus physiotherapy	Rotator cuff	Brox^36^	–	–	–	–	–	↔	–	–
	Rotator cuff	Ketola^29^	↔	–	–	–	–	↔	↔	
	Rotator cuff	Haahr^35^; Haahr^38^	↔	↔	–	–	–	↔	–	–
	Rotator cuff	Farfaras^28^	–	↑	↑	↔	–	↔	↔	↔
**Overall surgery versus physiotherapy (evidence level**)			**↔ (2**)	↑ (3)	↑ (3)	↔ (3)	- (4)	**↔ (2**)	**↔ (2**)	↔ (3)
Surgery versus ESWT	Rotator cuff	Rompe^30^	↔	–	–	–	–	↑		
	Wrist extensors	Radwan^31^	↔	–	–	↔	–	↔	–	–
**Overall surgery versus ESWT (evidence level**)			**↔ (2**)	– (4)	– (4)	↔ (3)	– (4)	↑ (3)	– (4)	– (4)
Surgery versus botox (evidence level)	Wrist extensors	Keizer^32^	↔ (3)	– (4)	↔ (3)	↔ (3)	– (4)	↔ (3)	– (4)	– (4)

Significant results (strong or moderate evidence) are highlighted in bold. Strong evidence (Level 1) is provided by generally consistent findings in multiple high-quality RCTs. Moderate evidence (Level 2) is provided by generally consistent findings in one high-quality RCT and one or more low-quality RCTs, or by generally consistent findings in multiple low-quality or moderate-quality RCTs. Limited or conflicting evidence (Level 3) is provided by only one RCT (either high or low quality), or by inconsistent findings in multiple RCTs. No evidence (Level 4) is defined by the absence of RCTs (van Tulder *et al*^26^, 2003).

ESWT, extracorporeal shock wave therapy; RCTs, randomised controlled trials.

#### Surgery versus no treatment/placebo

One good-quality study compared surgery with no treatment for shoulder tendinopathy. In the study by Beard *et al*,^34^ at 6-month and 12-month follow-up, the two surgical groups (corrective surgery and sham surgery) had a higher OSS than the no treatment group at statistical significance. A similar pattern was observed in the secondary outcomes, all of which had improved at 6 months in the corrective surgery group compared with the no treatment group. The modified Constant-Murley and HADS were statistically in favour of the sham surgery group compared with no treatment. At 12 months, the only significant difference was observed in the modified Constant-Murley score, which was higher in the two surgical groups compared with the no treatment group. Equally, patient satisfaction at 6 months was statistically higher in the two surgical groups versus the no treatment group; only some of the parameters were statistically significant at 12 months in favour of the surgical groups.

#### Surgery versus placebo (other than sham surgery)

One moderate-quality study compared surgery with placebo in patients with shoulder tendinopathy. Brox *et al*^36^ found that the detuned laser (placebo) group had a lower mean improvement in the Neer score and all its subcomponents compared with the two other treatment groups at 6 months and at this point the authors decided not to allocate more patients to the placebo group as it appeared to be inferior. Treatment success at 2.5-year follow-up was also in favour of the surgical group versus no treatment at statistical significance.

#### Surgery versus sham surgery

Two good-quality studies compared surgery with sham surgery. Kroslak & Murrell^22^ reported no statistically significant differences between the two groups in perceived pain, function and recovery at 6-month and >12-month follow-up. Both groups exhibited statistically significant improvements in self-rated pain frequency and severity, elbow stiffness and difficulty picking up objects at 6-month and >12-month follow-up as well as epicondyle tenderness, pronation-supination range, grip strength and modified ORI-TETS at 6-month follow-up compared with baseline. In the study by Beard *et al*^34^ at 6-month and 12-month follow-up, the two surgical groups (corrective surgery and sham surgery) had statistically higher OSS than the no treatment group.

#### Surgery versus physiotherapy

A total of six studies compared surgery with physiotherapy in shoulder tendinopathy (n=5) and patellar tendinopathy (n=1). Three of them were of moderate and three of poor overall quality. Brox *et al*^36^ were the first to compare surgery and any mode of conservative management with a randomised study in patients with shoulder tendinopathy. Comparing arthroscopic surgery and physiotherapy, there was a statistically insignificant difference in the Neer score improvement and pain reduction from moderate to mild favouring the surgical group. The latter outcome measure was found to be statistically significant when the comparisons were adjusted for sex (fewer females in the surgical group at baseline) in favour of the surgical group. At 2.5-year follow-up, success rates (defined as Neer score >80) were similar between those who received exercises only and those who received surgery.

In a similar study in patients with shoulder tendinopathy, Haahr *et al*^35^ reported no differences in Constant score (primary outcome) and its sub-scores (pain, function, ROM, force) between their two groups over 1 year. Differences in the secondary outcomes (pain and dysfunction) were also non-significant at 1-year follow-up. Six of the patients in the physiotherapy group (14%) ended up having an operation within the 12 months; comparisons at 12 months were performed as per ‘intention-to-treat’ which may have resulted in results being biassed in favour of the physiotherapy group. The same group^38^ later found no significant differences between the two groups in terms of income transfers, obtaining a disability pension 4 years after inclusion and self-reported outcomes as measured by the PRIM score 4–8 years after inclusion.

Rahme *et al*,^37^ in their study of shoulder tendinopathy, investigated surgical patients receiving postoperative physiotherapy, the nature or further details of which are not reported; therefore, we do not consider this as combined treatment. Even though the emphasis of the study was on predictive factors and pain-generating mechanisms, at 6-month follow-up there was no difference in the two groups with regard to the proportion who had achieved at least 50% reduction of the initial total pain score. After the 6-month time point, more than half of the physiotherapy group were given the opportunity and elected to have surgery and results at 12-month follow-up are presented on an ‘as treated’ and not on an ‘intention-to-treat’ basis.

In their study, Ketola *et al*^29^ found no differences between patients with shoulder tendinopathy receiving physiotherapy versus those receiving physiotherapy plus surgery in the primary (self-rated pain) or secondary (disability, night pain, SDQ score, number of painful days, proportion of pain-free patients) outcomes at 2- and 5-year follow-up. Both groups demonstrated statistically significant differences in all outcome measures at 5-year follow-up compared with baseline.

In another shoulder tendinopathy study by Farfaras *et al,*^28^ both surgical groups (open and arthroscopic) received the same physiotherapy regime as the physiotherapy only group postoperatively. Compared with baseline, none of the three treatment groups demonstrated significant differences in the overall SF-36 score at follow-up (mean 31 months) with no intergroup differences. All three groups improved significantly in terms of internal rotation at follow-up versus baseline with no significant difference between groups. The Constant score improved at statistical significance from baseline to follow-up in the two surgical groups but not in the physiotherapy group; however, no significant intergroup differences were observed. Active elevation strength only improved significantly in the open surgery group at follow-up compared with baseline but, similarly, the three groups were statistically similar at follow-up. The same group reported results of the same patients at >10-year follow-up which favour surgery over physiotherapy. The surgical groups demonstrated significantly improved active elevation ROM compared with the physiotherapy group, internal rotation improved within all groups from baseline to follow-up but not between groups and muscle strength only improved significantly at follow-up within the open surgery group without intergroup differences.

In the study by Bahr *et al*^27^ in patellar tendinopathy, VISA score improved significantly in both groups with time; however, there was no statistically significant differences between the groups at any stage of follow-up. Similarly, there were improvements in the leg-press strength test with time in both groups but no intergroup differences. Jump height did not change in either group at any stage of follow-up compared with baseline and the two groups were statistically similar. Compared with baseline, pain scores during functional tests improved at 12 months but not 6 months in both groups and there were no differences between groups. Equally, there was no difference in overall treatment satisfaction or return to sports between groups at 12 months. Finally, with respect to the global evaluation score, the eccentric group demonstrated improved outcomes at statistical significance compared with the surgical group at 3 months; however, the two groups were statistically similar at 6 and 12 months.

#### Surgery versus ESWT

One poor-quality study and one moderate-quality study compared the effectiveness of (open) surgery versus ESWT in chronic tendinopathy. Rompe *et al*^30^ tested the two modalities in patients with shoulder tendinopathy and reported improved clinical outcome in terms of the UCLA score in the surgical group versus the ESWT group at 24 months follow-up. Self-rated pain reduction at 24-month follow-up was similar between the two groups. Finally, hospital stay and absence from work were significantly shorter in the ESWT group.

In the study by Radwan *et al*,^31^ patients with lateral elbow tendinopathy treated surgically exhibited no significant differences in any of the outcome measures compared with those receiving ESWT at any of the follow-up stages. Significant improvements with time were observed in all outcome measures in both treatment groups.

#### Surgery versus botox

One poor-quality study compared surgery with botox injections in chronic lateral elbow tendinopathy.^32^ In terms of overall results and pain, the two treatment groups were statistically comparable at all follow-up stages. Compared with the botox group, the surgical group exhibited a greater extension deficit at 3 and 6 months but the difference had disappeared at 12 and 24 months. Sick leave was significantly shorter in the surgical group versus the botox group at 3 months; however, no statistically significant longer-term differences were observed.

#### Surgery versus polidocanol

One poor-quality study allocated patients with Achilles tendinopathy to either surgery (colour Doppler-guided) or polidocanol injections.^33^ At 12-week follow-up, 67% of the patients in the polidocanol group and 80% of those in the surgical group were satisfied with the results and returned to their pre-injury recreational/sport activity (statistical comparison not presented). Pain scores reduced at statistical significance in both groups compared with baseline and even though no between-group statistical comparisons are presented, pain improvement at 12 weeks appears to be similar in the two groups (VAS scores 76 to 24 in polidocanol group and 75 to 21 in surgical group). At 6 months, 100% of the surgical group versus 67% of the polidocanol group were satisfied with treatment and returned back to their pre-injury recreational/sport activities; again, no statistical comparisons are reported.

## Discussion

We found no evidence for superiority of surgery to exercise-based therapies in patients with tendinopathy. To our knowledge, this is the first systematic review comparing surgery with no treatment, sham surgery and exercise-based therapies modalities in all tendinopathies.

Some studies advocate surgery for tendinopathies after 3–6 months of conservative management.^27 36^ Our analysis demonstrates that outcomes after tendon loading exercises both up to 12 months and longer term are as good as surgery, at least for shoulder tendinopathy. An interesting finding of our review is that surgery appeared to be superior to no treatment or placebo but not to sham surgery. While the placebo group that received detuned laser in the study by Brox *et al*^36^ exhibited no improvement in the Neer shoulder score at 6-months follow-up, the group of patients that received no treatment in the study by Beard *et al*^34^ had a higher OSS at both 6 and 12 months compared with baseline.

This discrepancy may be a result of different outcome measures and/or sample sizes in the two studies or other methodological differences. Regardless of this discrepancy, surgery was significantly more effective than detuned laser and no treatment in the two studies but not to sham surgery in the latter study. This is in accordance with the findings of Kroslak & Murrell^22^ who found no differences in outcomes with the Nirschl procedure versus sham surgery in patients with lateral elbow tendinopathy. According to Beard *et al,*^34^ the difference between surgery and no treatment, taking into account the similar effects of arthroscopic decompression and sham arthroscopy, may be attributable to surgical placebo effect, unidentified effects of arthroscopic assessment of the joint and bursa, and rest and postoperative physiotherapy associated with surgery. Based on their findings, the authors state that arthroscopy (with or without decompression) could be used for the treatment of shoulder tendinopathy but at the same time they suggest assessing other management strategies apart from surgery.

Sham surgery in randomised controlled surgical trials is gaining increasing popularity despite ethical considerations and studies with sham surgery in orthopaedics have reported interesting results.^23 39^ Compared with using a non-surgical control group, sham surgery equalises the placebo effect of surgery and can give more realistic insights into the effectiveness of the actual surgical procedure in question.^40^ In their recent systematic review of sham surgery in orthopaedics, Louw *et al*^41^ included six studies comparing orthopaedic procedures with sham surgery, one of which was the study by Kroslak & Murrell^22^ included in the present review. The authors concluded that sham surgery appears to be as effective as corrective surgery in terms of pain and disability for certain conditions; however, the results are not necessarily generalisable to operations not included in the review. This is in accordance to our study, which additionally showed similar outcomes of sham and corrective surgery in function and ROM in shoulder tendinopathy and lateral elbow tendinopathy. The exact mechanisms of surgery (corrective or sham) leading to improvement of outcomes in tendinopathy remain uncertain and the possibility of this improvement being due to the postoperative tendon rehabilitation cannot be ignored.

Despite the rigour of our review with respect to identifying all the available evidence and the quality assessment of the included studies, we recognise study limitations. First, due to the small number of eligible studies and the different comparisons of surgery with each non-surgical treatment modality, our conclusions on most outcomes had a poor level of evidence. Equally, due to the lack of adequate data, different tendinopathies were clustered together in some comparisons (surgery vs sham surgery; surgery vs ESWT; surgery vs physiotherapy) to increase the strength of evidence. Although we acknowledge this as a potential drawback of our study, we expect specific treatments may potentially yield to similar (if not identical) effects on tendinopathies at different sites as they share the same pathophysiology. However, we did not generalise conclusions on comparisons of modalities to include types of tendinopathy that did not contribute any results for that specific comparison. Additionally, the wide range of outcome measures used by authors resulted in lack of homogeneity which made the conduction of a meta-analysis impossible. The different regimes and intensities of physiotherapy and postoperative rehabilitation used in studies might have affected the results and, in patients treated surgically, the possibility of improvement due to the postoperative rehabilitation/physiotherapy cannot be overlooked. Due to the small patient numbers in many of the studies, our inability to calculate a minimal clinically important difference may mask the fact that statistically significant differences differ from ultimate meaningful benefit to these patients with tendinopathy. Finally, as the duration of symptoms of tendinopathy in some studies^27 29 36^ was only 3 months, natural progression of the disease may have improved patient outcomes.

## Conclusion

In this systematic review of 12 eligible RCTs in patients with various tendinopathies, surgery was not superior to sham surgery in patients with tendinopathy in the midterm and long term. Further well-designed randomised studies with large populations comparing surgery with both tendon loading regimes and sham surgery are warranted. In the meantime, we advocate that healthcare professionals who treat patients with tendinopathies should reserve surgery for selected cases and only after a sufficiently long course (12 months) of evidence-based loading exercise has failed.
